# Uni-center, patient-blinded, randomized, 12-month, parallel group, noninferiority study to compare outcomes of 3-row vs 2-row circular staplers for colorectal anastomosis formation after low anterior resection for rectal cancer

**DOI:** 10.1097/MD.0000000000015978

**Published:** 2019-06-14

**Authors:** Nikita A. Nekliudov, Petr V. Tsarkov, Inna A. Tulina

**Affiliations:** a Sechenov Biomedical Science and Technology Park, Sechenov First Moscow State Medical University; b Department of Surgery, Faculty of Preventive Medicine, Clinic of Colorectal and Minimally Invasive Surgery, Sechenov First Moscow State Medical University, Moscow, Russian Federation.

**Keywords:** anastomotic leakage, colorectal anastomosis, colorectal neoplasms, low anterior resection, rectal cancer, stapled anastomosis, surgical stapling

## Abstract

**Background:**

Colorectal anastomotic leakage (AL) is one of the most serious complications in rectal cancer surgery due to its negative impact on the overall as well as cancer-specific survival. Two-row stapling technique has become standard in low anterior resections (LARs), but has neither alleviated the morbidity, nor reduced the incidence of AL. This is the 1st study that aims to compare the success rate of new 3-row circular staplers compared to that of conventional 2-row staplers in a prospective, randomized clinical trial.

**Methods:**

The THREESTAPLER trial (Clinical Trials NCT03910699) is a prospective, noninferiority, 2-armed, parallel-group, patient and outcomes assessor blinded study with a 1:1 allocation ratio. Colorectal anastomosis will be formed using Ethicon 29 mm Curved Intraluminal Stapler (CDH29A) in the active comparator group, and using Mirus Disposable Circular Stapler 3 Row 29 (MCS-29R3) in the experimental group. The hypothesis states that the incidence of AL in the 3-row stapler group is at least not higher than in the 2-row stapler group. Assuming there is a difference in success rate of 12% and noninferiority margin Δ = 5%, 154 patients will be required to achieve statistical significance. An interim analysis will be performed after recruitment of 20 patients per group to assess safety profile of 3-row circular staplers. The primary endpoint is the rate of AL, documented by imaging studies, assessed with Pearson Chi-squared test and Fisher exact test. The secondary outcomes include severity of AL (A, B, or C), anastomotic bleeding, postoperative complication rate graded with the Clavien–Dindo classification, reintervention rate, stapler dysfunction rate, complications of defunctioning stoma, overall and cancer-specific quality of life, assessed with short form (36) and quality-of-life questionnaire core 30 questionnaires, respectively, fecal incontinence assessed with Cleveland clinic incontinence score form, and manifestation of LAR syndrome. All patients will be monitored for 12 months following the LAR.

**Discussion:**

This is the 1st prospective randomized trial to assess the safety profile of 3-row staplers for colorectal anastomosis after LAR for rectal cancer. It may provide evidence of feasibility of 3-row circular staplers in LAR with respect to short-term and long-term patient outcomes.

Trial registration: NCT03910699 on 10 April 2019

## 1 Introduction

### 1.1 Background and rationale

Anastomotic leakage (AL) is one of the most serious complications of low anterior resection (LAR) with a sphincter-preserving total mesorectal excision (TME) for rectal cancer. Despite the refinement of the surgical techniques used in rectal cancer surgery, AL rate remains high with a reported value in a recent systematic review of 6.3%.^[1]^ However, a retrospective study assessing the rate of AL after LAR with double-stapling technique (DST) has reported AL rate of 12.3%.^[2]^ Additionally, a 2010 systematic review that included a broader patient population has demonstrated AL rate of 11%.^[3]^ Thus, the incidence of AL varies widely as it has a multifaceted etiology due to a large number of risk factors. AL has a detrimental effect on both short-term and long-term patient outcomes due to high reintervention and a decreased survival rate. A recent systematic review has shown an association between AL and a higher chance of local cancer recurrence, as well as with lower overall and cancer-specific survival.^[4]^ The increase in cancer recurrence can be explained by “extraluminal tumor implantation” by free malignant cells that may be present in anastomotic line. AL is also associated with higher costs due to high reintervention rate: according to retrospective national cohort study conducted by Boström et al in Sweden that included 6948 patients within the period from 2007 to 2016, 693 of patients (10.0%) experienced AL, and 294 (4.2%) underwent reintervention due to AL.^[5]^

Two-row staplers have long been standard in colorectal in general surgery; however, 3-row stapling devices have also emerged. Although circular staplers have simplified low and ultra-low anastomoses formation,^[6]^ their usage did not decrease the risk of AL. Moreover, the efficacy and safety of 3-row staplers has only been briefly addressed in the existing literature.^[7,8]^ Previous studies have almost exclusively focused on comparison of different techniques of colorectal anastomosis formation,^[9,10]^ whereas the research assessing different types of stapled anastomoses is mostly limited to single- vs double-stapled techniques.^[11,12]^ Up to date, no previous research has investigated the efficacy of 3-row circular staples for colorectal anastomoses formation compared to that of conventional 2-row staplers. However, retrospective observational study by Foo et al has compared the outcomes of 3- and 2-row linear stapler usage for ileocolic anastomoses formation. Notably, the reoperation rate in 3-row staplers group was found to be significantly lower in the experimental group (5.9%) compared to 1.8% in the control group *(P* *=* .049).^[7]^ Thus, a lower morbidity rate was demonstrated in tri-staplers group. Despite the indefinite results, the findings of the study suggest further need for evaluation of efficacy of 3-row staplers for low colorectal anastomoses formation in a prospective randomized controlled clinical trial (Table 1).

**Table 1:**
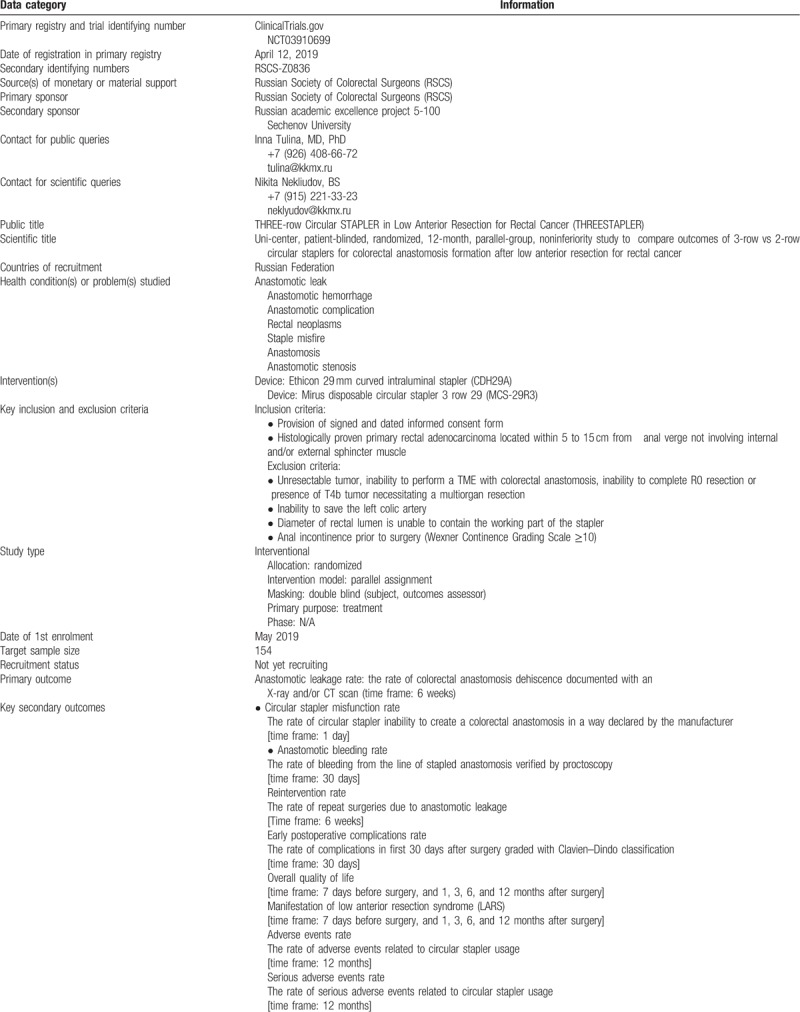
Data set.

The data on comparison of different types of circular staplers for colorectal anastomosis formation are scarce. The results of a Swedish trial suggest that the type of stapler is an independent risk factor of AL.^[13]^ Therefore, our study aims to investigate safety and efficacy of 3-row circular stapler (Mirus Disposable Circular Stapler 3 Row 29 [MCS-29R3]) as compared to standard 2-row circular stapler (Ethicon 29 mm Curved Intraluminal Stapler [CDH29A]) that have been conventionally used in LARs for over 10 years. All foreseeable patient risks are attributed the operation remain similar in both control and experimental groups. Possible anastomosis-related complications include anastomotic leak, anastomotic bleeding, anastomotic stricture, para-anastomotic abscess.

### 1.2 Objectives

1. Research hypothesis: 3-row staplers are noninferior to 2-row staplers in colorectal anastomosis formation with respect to anastomotic leakage rate, meaning that AL rate in 3-row-stapled colorectal anastomoses is at least not higher than that of 2-row-stapled anastomoses within 5% noninferiority margin.
2. Study objectives
  2.1. Primary objective: to determine if 3-row staplers are noninferior to 2-row staplers (noninferiority margin Δ = 5%) in the endpoint of anastomotic leakage rate determined by X-ray or computed tomography (CT) scan in subjects undergoing LAR for rectal cancer.
  2.2. Secondary objectives
    2.2.1. Key secondary objectives
      2.2.1.1. Evaluate the incidence of anastomosis-related complications, including anastomotic bleeding and para-anastomotic abscess formation
      2.2.1.2. Determine the impact of the stapler type on overall and cancer-related quality of life using short form (36) (SF-36) and quality-of-life questionnaire core 30 (QLQ-C30) questionnaires, respectively
    2.2.2. Other secondary objectives
      2.2.2.1. Assess the incidence of LAR syndrome (LARS) and level of incontinence during postoperative period in patients with stapled colorectal anastomosis.
      2.2.2.2. Determine the impact of the stapler type on incontinence, assessed with the Cleveland clinic incontinence score (CCIS) questionnaire.

### 1.3 Trial design

THREESTAPLER study is a prospective, randomized, controlled, patient and outcomes assessor blinded, uni-center, noninferiority, parallel group, 2-arm trial with 1:1 allocation ratio. Randomization will be performed using block method after intraoperative eligibility assessment.

## 2 Methods

### 2.1 Study setting

The study will be conducted at the Clinic of Colorectal and Minimally Invasive Surgery of the Sechenov University Hospital N. 2 in Moscow, Russia. Patients with colorectal cancer comply a significant part of patient body; on average, 150 patients are diagnosed with and treated for rectal cancer per year, the interventions include open, laparoscopic, and robotic approaches. Thus, in this case, a single-center design can assure sufficient patient recruitment.

### 2.2 Eligibility criteria

To be eligible for randomization, an individual must comply with all of the following criteria:

Inclusion criteria

1.1. Provision of signed and dated informed consent form
1.2. Stated consent to comply with all study procedures and availability for the duration of the study
1.3. Male or female
1.4. For females of reproductive potential: not pregnant at the time of screening
1.5. For males of reproductive potential: use of condoms or other methods to ensure effective contraception with partner
1.6. Histologically proven primary rectal adenocarcinoma located within 5 to 15 cm from anal verge not involving internal and/or external sphincter muscle

Exclusion criteria

2.1. Preoperative and intraoperative
  2.1.1. Current use of antiplatelet drugs, acetylsalicylic acid, or anticoagulants within 7 days prior to intervention
  2.1.2. Unresectable tumor, inability to perform a TME with colorectal anastomosis, inability to complete R0 resection, or presence of T4b tumor necessitating a multiorgan resection
  2.1.3. Inability to save the left colic artery
  2.1.4. Diameter of rectal lumen is unable to contain the working part of the stapler
  2.1.5. Infection requiring antibiotic treatment within 30 days prior to intervention
  2.1.6. Anal incontinence prior to surgery (Wexner continence grading scale ≥10)

Postoperative

2.2.1. Inability to complete all the trial procedures
2.2.2. Death due to causes unrelated to anastomotic leak in early postoperative period
2.2.3. Patient wants to withdraw from the clinical trial

### 2.3 Interventions

#### 2.3.1 Screening

If the patient assigned an elective low anterior rectal resection meets the inclusion criteria, the patient will be asked to review the informed consent form for participation in the study; a detailed explanation of the participant's obligations and rights in case of participating in the study will be provided. All interventions related to the study will be carried out only if the patient has signed the informed consent. Upon provision of the informed consent, the patient will be assigned a number of procedures aimed at assessing the patient's compliance with the eligibility criteria:

1. Sigmoidoscopy
2. Tumor biopsy
3. Computed tomography of the abdominal cavity and chest
4. MRI of the pelvis
5. Internist and anesthesiologist consults
6. Oncology case conference
7. Baseline assessment of the overall and cancer-specific quality of life (SF-36 and QLQ-C30 questionnaires, respectively)
8. Incontinence assessment (CCIS)

Based on the results of the screening, the stage of the disease, indications and type of surgical treatment, the possibility of performing a TME, and surgical approach (laparoscopic or open) are determined.

#### 2.3.2 Preoperative preparation

On the day before the surgery, mechanical bowel preparation is performed by oral administration of polyethylene glycol or sodium phosphate solution. The standard premedication regimen includes phenazepam 0.5 mg at night before bed and morning before surgery. Depending on present comorbidities, an individual regimen of preoperative examination and preparation may be selected.

#### 2.3.3 Day of surgery

Under a combined general anesthesia, the patient is placed in a modified lithotomy position on the back, with legs spread apart on supports and arms along the torso. The operative field is treated with an antiseptic solution twice and draped.

##### 2.3.3.1 Open approach

A midline laparotomy is performed. After an examination of the abdominal cavity and the pelvic cavity, mobilization of the sigmoid colon in the lateral-medial direction begins. Para-aortic lymph node dissection is performed and inferior mesenteric artery is skeletonized with preservation of the integrity of preaortic and prehypogastric plexuses. The inferior mesenteric artery is ligated below the origin of the left colic artery; the latter is preserved. The inferior mesenteric vein is cut at the level of the superior edge of the body of the pancreas. The mesentery of the sigmoid colon is cut in the direction of the intestinal wall. The place of the transection of the sigmoid colon is determined at a distance of at least 15 cm above the tumor in an area with an adequate blood supply. Sigmoid colon is divided and the circular stapler anvil is fixed in its stump with a purse-string suture. After that, a TME is performed with the preservation of the hypogastric nerves and pelvic nerve plexuses. L-shaped clamp is applied to the rectum at least 2 cm below the tumor. From the side of the perineum, the rectal stump is washed with an antiseptic solution. After that, below the L-shaped clamp, a linear cutter stapler is applied and the rectum is transected. The surgeon inserts the circular suturing device into the anal canal. Under the guidance of a direct observation, the trunk of the operating part of the device is pierced in the area of one of the edges of the suture line of the rectal stump. The stump of the sigmoid colon is moved to the pelvic cavity, the rod of the device anvil is put on the trunk of the operating part, and end-to-end colorectal anastomosis is formed. The height of the anastomosis is measured (in centimeter from the edge of the anal canal). After the removal of the circular suturing device, the integrity of the resected intestinal rings is checked. The suture line is tested for leakage. A transanal silicone tube is installed. A preventive transverse colostomy is formed. A silicone drain tube is installed in the pelvic cavity. The laparotomy wound is sutured. Sterile dressings are applied.

##### 2.3.3.2 Laparoscopic approach

Following trocar installation and examination of abdominal cavity and pelvic cavities, para-aortic lymph node dissection is performed with the skeletonization of the inferior mesenteric artery and preservation of the integrity of the preaortic and prehypogastric plexuses. The inferior mesenteric artery is divided below the origin of the left colic artery; the latter is preserved. The sigmoid colon and the distal portion of the descending colon are mobilized in the medial-lateral direction. The inferior mesenteric vein is cut at the level of the upper edge of the body of the pancreas. After that, a TME is performed with the preservation of the hypogastric nerves and pelvic nerve plexuses. The rectum is transected with a linear cutter stapler at least 2 cm below the inferior margin of the tumor. A mini-laparotomy is performed. In the wound, the separated rectum and the sigmoid colon are removed, the mesentery of the sigmoid colon is transected in the direction of the transection of the intestinal wall. Sigmoid colon is divided and the circular stapler anvil is fixed in its stump with a purse-string suture. The stump of the intestine with circular suturing device anvil is immersed in the abdominal cavity, the laparotomy wound is sutured. A repeated laparoscopy is performed. From the side of the perineum, after preliminary washing of the rectal stump, the surgeon inserts the circular suturing device into the anal canal. Under guidance with a laparoscope, the trunk of the working part of the apparatus is pierced in the area on one of the edges of the suture line of the rectal stump. The sigmoid colon stump is moved to the pelvic cavity, the rod of the device head is inserted into the trunk of the operating part, an end-to-end colorectal anastomosis is secured. The height of the anastomosis is measured in centimeter from the anal verge. After the removal of the circular suturing device, the integrity of the resected intestinal rings is assured. The suture line is tested for leakage. A transanal silicone tube is installed. A preventive transverse colostomy is formed. A silicone drain tube is installed in the pelvic cavity. Abdominal wall openings are sutured. Sterile dressings are applied.

#### 2.3.4 Early postoperative period

It is defined as the time period from the completion of the surgery and up to 30 days after the surgery. The patient is extubated after return of consciousness, reflexes and muscle tone, either in the operating room, or the intensive care unit (ICU). If necessary, the patient may require a prolonged mechanical ventilation. In the ICU, vital functions are monitored, with a special attention paid to the drainage discharge and the state of postoperative dressings. An individual anesthesia regimen is selected, which may include NSAIDs, opioid pain relievers, and regional block anesthesia. Laboratory tests (usually twice a day, more often if necessary), correction of electrolyte imbalance, prokinetic agents, prevention of thromboembolic complications, antibiotics and PPIs are mandatory. Intake of colorless liquid is permitted from the 1st hours after surgery, the volume of liquid consumed per day depends on time of return of bowel function and the extent of the surgery. Parenteral nutrition may be prescribed if the absence of enteral nutrition within 72 hours is expected, or due to baseline protein-energy malnutrition. The treatment is also adjusted to the existing comorbidities.

Patient ambulation occurs on the 1st day, routinely in the morning of the next day postsurgery. The patient is put into upright position without sitting down. Upon compensation of the vital functions and the absence of foreseen complications of nonsurgical nature, the patient is transferred from the ICU to ward, where urinary catheter is removed.

#### 2.3.5 Late postoperative period

It is defined as the time period starting at 31st day postsurgery. At weeks 4 to 6, according to the standard protocol, the preventive stoma is closed. Also, the patient is asked to fill out the questionnaire forms SF-36, QLQ-C30, LARS, and CCIS to assess the changes in the quality of life of the patient relative to the baseline level.

Following discharge, all subjects will be required to provide 4 filled out questionnaires (SF-36, QLQ-C30, CCIS, LARS), and report their pain level using the VAS. To ensure patient compliance to protocol requirements, all subjects will be notified telephonically and/or via e-mail. If the patient is unavailable, he or she must be contacted at least 3 times within the follow-up timeframe, before considered dropped-out from the study.

All subjects are strongly advised to adhere to bland diet, and are recommended to abstain from the intake of meat and fatty meals within at least 1 month after stoma reversal. Patients are also advised to minimize the intake of NSAIDs in the absence of comorbidities requiring NSAID treatment. NOAC administration is necessary during the 1st week after surgery to minimize the risk of thrombosis.

### 2.4 Outcomes

#### 2.4.1 Primary outcome measure

The primary outcome measure is the difference between the 2 groups in the incidence of colorectal anastomosis leakage documented with an X-ray and/or CT scan in the time period between the LAR and stoma reversal surgery.

As proposed by the International Study Group of Rectal Cancer, AL is defined as a “defect of the intestinal wall integrity at the colorectal or coloanal anastomotic site (including suture and staple lines of neorectal reservoirs) leading to a communication between the intra- and extraluminal compartments,” or presence of “a pelvic abscess close to the anastomosis.”^[14]^ In context of primary outcome analysis only presence or absence of diagnosed AL (dichotomous aggregation method) from the day of intervention to preventive stoma reversal will be considered. The timeframe for the primary outcome measure is defined as the period between the initial surgery and stoma reversal, which usually takes place at 4 to 6 weeks after LAR, and may differ significantly between subjects due to variability of AL presentation and symptoms. Additionally, AL may be asymptomatic and discoverable only with imaging studies, which are only necessary to ensure the safety of stoma reversal surgery.

All patients undergoing LAR with stapled colorectal anastomosis formation will be treated according to intervention protocol described in the corresponding section. The patients’ status will be monitored twice a day during rounds. AL has a multifaceted clinical course and has no pathognomonic features; it may be asymptomatic and require no intervention, or it can lead to serious complications, such as peritonitis and sepsis. The clinical signs of AL include the following:

1. General
  - 1.1. Fever (≥38.0°C)
  - 1.2. Tachycardia (≥100/min)
  - 1.3. Increased respiratory rate (≥30/min)
  - 1.4. Renal insufficiency (<30 mL/h or <700 mL/d)
  - 1.5. Altered mental status (agitation/lethargy, GCS < 15)
  - 1.6. Deterioration of overall clinical condition
  - 1.7. Peritonitis (clinical signs of abdominal guarding, rebound tenderness, abdominal pain worsening with movement, etc)
  - 1.8. Sepsis (qSOFA ≥ 2)
2. Local
  - 2.1. Bowel obstruction
  - 2.2. Abnormal drain content (purulent/turbid/air)
  - 2.3. Purulent or fecal vaginal discharge (rectovaginal fistula)
  - 2.4. Abdominal pain other than wound pain
  - 2.5. Fascial dehiscence
3. Laboratory signs include:
  - 3.1. Leukocytosis
  - 3.2. C-reactive protein elevation

**Figure 1:**
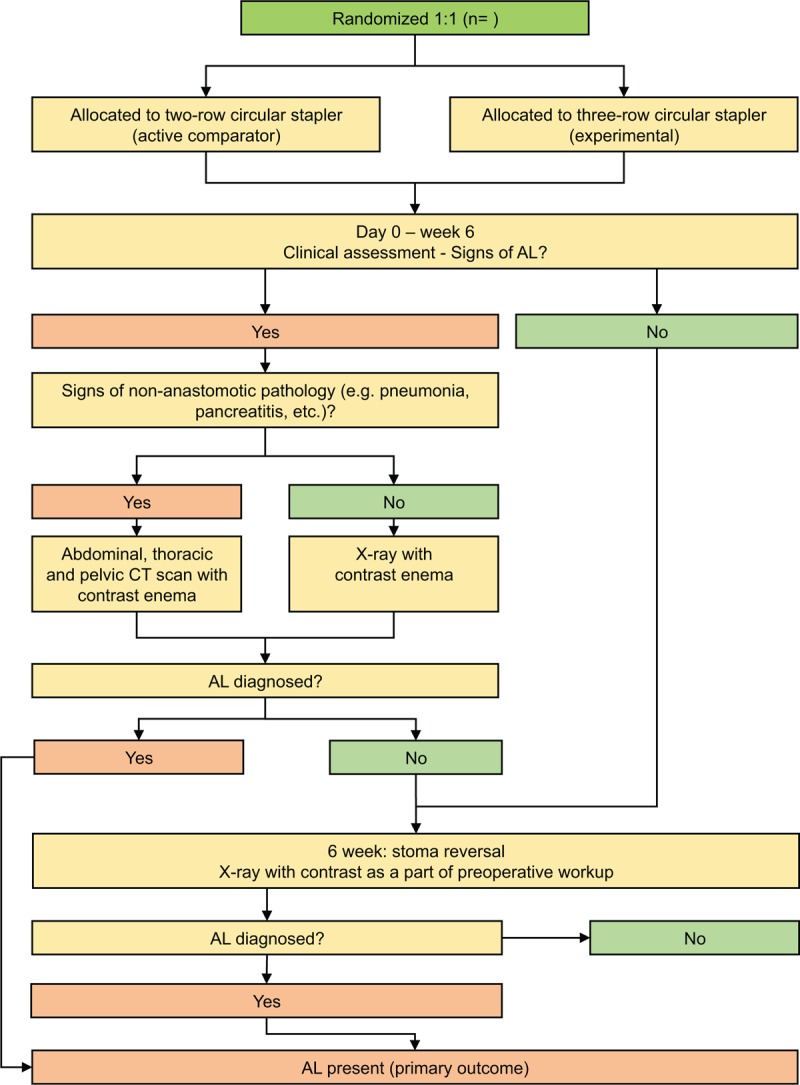
Primary outcome measure. AL *=* anastomotic leakage, CT *=* computed tomography.

There are several ways in which AL can be diagnosed in the investigated subjects (Fig. 1).

1. If any of these clinical signs prompting the attending surgeon to suspect AL are present, the patient will undergo imaging studies for further evaluation. If there is no evidence of nonanastomotic complications, an X-ray with contrast enema will be performed. In presence of any clinical signs of nonanastomotic complications (pancreatitis, pneumonia, etc), a CT scan of the chest, abdomen, and pelvis with contrast enema will be obtained to simultaneously exclude anastomosis-unrelated postoperative complications. If CT results suggest AL, the patient will reach the primary endpoint, and will be treated accordingly to their condition. Further treatment will not be discussed in this protocol as it is irrelevant to endpoint analysis.
2. If there are no symptoms suggesting AL before discharge, the subject will undergo X-ray with contrast enema as a part of preoperative assessment prior to stoma reversal procedure in 4 to 6 weeks after the initial surgery according to the standard protocol implemented at the study center. If the X-ray findings suggest AL, the subject will reach the primary endpoint.

#### 2.4.2 Secondary outcome measures

1. Severity of AL (A, B, and C): According to the grading system suggested by Rahbari et al in the aforementioned study,^[14]^ AL is classified into^[3]^ stages depending on the necessity of therapeutic or surgical intervention.
  - Grade A: no active therapeutic intervention required
  - Grade B: requires active therapeutic intervention, but manageable without relaparotomy
  - Grade C: requires relaparotomy
2. Operating time (time frame: 1 day): The duration of surgical procedure in minutes.
3. Circular stapler dysfunction rate (time frame: 1 day): The rate of circular stapler inability to create a colorectal anastomosis in a way declared by the manufacturer.
4. Anastomotic bleeding rate (time frame: 30 days): The rate of bleeding from the line of stapled anastomosis verified by proctoscopy.
5. Reintervention rate (time frame: 6 weeks): The rate of repeat surgeries due to anastomotic leakage.
6. Early postoperative complications rate (time frame: 30 days): The rate of complications in 1st 30 days after surgery, Clavien–Dindo postoperative complication grades I to V.
7. The postoperative hospital stay (time frame: 1 month): The number of days from the 1st day after operation to discharge.
8. Complications of defunctioning stoma (time frame: 3 months): The rate of complications related to defunctioning stoma.
9. Overall quality of life (time frame: 7 days before surgery, and 1, 3, 6, and 12 months after surgery): Assessed with patient-reported questionnaire SF-36. A total score in each of 8 sections will be calculated and transformed into a 0 to 100 scale with a score of 0 equivalent to maximum disability and a score of 100 equivalent to no disability.
10. Cancer-related quality of life (time frame: 7 days before surgery, and 1, 3, 6, and 12 months after surgery): Assessed with patient-reported questionnaire European Organization for Research and Treatment of Cancer (EORTC) QLQ-C30 with supplementary module EORTC QLQ-CR29. A total score in each of 4 modules (functional scale, global health status, symptom scale, colorectal cancer module) will be calculated and converted into a 0 to 100 scale. For functional scale, global health status scale, and colorectal cancer module a score of 0 is equivalent to maximum disability and a score of 100 is equivalent to no disability. For symptom scale a score of 100 is equivalent to maximum disability and a score of 0 is equivalent to no disability.
11. Manifestation of LARS (time frame: 7 days before surgery, and 1, 3, 6, and 12 months after surgery): Assessed with patient-reported questionnaire LARS, total score will be calculated (minimum 0, maximum 42) for each patient and also each patient will be assigned to either “no LARS” group (total score 0–20), “minor LARS” group (total score 21–29), or “major LARS” group (total score 30–42).
12. Manifestation of fecal incontinence (time frame: 7 days before surgery, and 1, 3, 6, and 12 months after surgery): Assessed with patient-reported questionnaire CCIS, total score will be calculated (minimum 0, maximum 20) for each patient, where 0 means perfect continence and 20, complete incontinence.
13. Adverse events rate (time frame: 12 months): The rate of adverse events related to circular stapler usage.
14. Serious adverse events rate (time frame: 12 months): The rate of serious adverse events related to circular stapler usage.

### 2.5 Participant timeline

**Figure 2:**
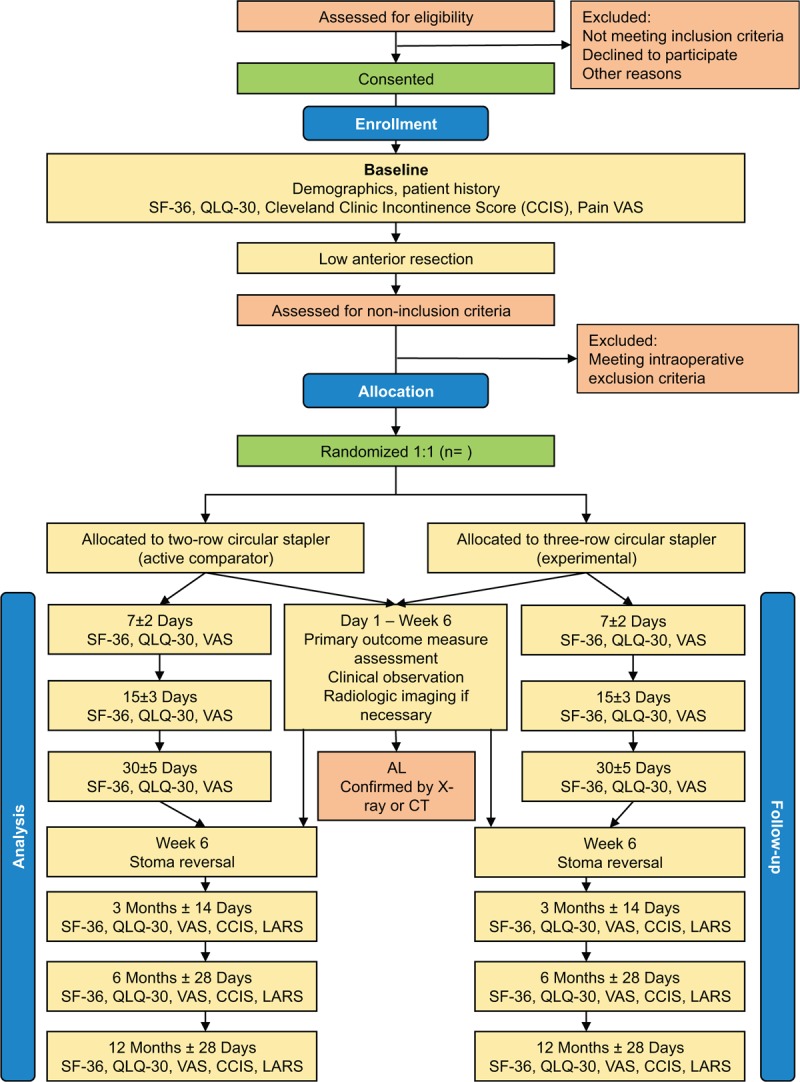
Participant timeline. AE & SAE = adverse events & serious adverse events, CCIS = Cleveland clinic incontinence score, H&P = history and physical examination, LARS = low anterior resection syndrome (questionnaire), QLQ-C30 = quality-of-life questionnaire core 30, QoL = quality of life, SF-36 = short form (36 item), VAS = visual analog scale.

A flowchart of participant timeline is shown in Figure 2.

### 2.6 Sample size

Considering that this is a noninferiority study, the sample size was calculated using 1-sided Blackweder test.^[15]^ According to published data, the incidence of colorectal anastomotic leakage formed standard DST varies from 7% to 25%.^[16]^ Colorectal AL rate after LAR with preventive stoma formation at the study center averages at 18%. The expected incidence of colorectal AL after 3-row suturing device is 30%. The purpose of this study is to obtain preliminary results demonstrating that the outcomes of colorectal anastomosis formation with a 3-row suturing device are noninferior compared to those of a 2-row suturing device. Considering that *α* = 0.05; the statistical power of the study is 80%; the patients are randomized into 2 groups with 1:1 allocation ratio; the noninferiority margin Δ = 5%, the required sample size is 154 patients (77 patients in each of the 2 groups). Thus, if there is a true difference in favor of the experimental treatment of 12% with respect to AL rate, then 154 patients are required to be 80% sure that the upper limit of a 1-sided 95% confidence interval will exclude a difference in favor of the standard group of more than 5%.

In order to conduct an interim analysis to assess the safety of 3-row suturing devices for the formation of a colorectal anastomosis, 20 patients will be included in each group. Given a 10% probability of patients leaving the study, it is necessary to recruit 22 patients in each group. Thus, a sample of 44 patients (22 in each of the 2 comparison groups) should allow for a reliable preliminary assessment of the safety and feasibility of the use of 3-row suturing devices for the formation of colorectal anastomosis.

### 2.7 Recruitment

All patients diagnosed with rectal cancer confirmed with imaging studies and colonoscopy with biopsy, assigned an elective LAR will be considered for this study. In case of matching all the inclusion criteria, and upon provision of consent form signed by the patient, or legal guardian, the patient will be asked to fill out SF-36 and QLQ-C30 questionnaires for baseline quality of life assessment. The patients will also be required to fill out the CCIS questionnaire for baseline assessment of previous incontinence.

### 2.8 Assignment of interventions

#### 2.8.1 Allocation

Participants will be randomly assigned to either control or experimental group with a 1:1 allocation ratio using fixed block randomization with a computerized random number generator. The block size equals at least 4 and will be unknown to the principal investigator, outcomes assessor and the operating surgeon. Because the possibility of making a stapled anastomosis will only be determined intraoperatively by the operating surgeon with respect to exclusion criteria precluding stapled anastomosis formation, all subjects will be allocated intraoperatively.

#### 2.8.2 Blinding

All relevant data from patient chart except patients’ names and the stapler used during surgery will be transferred into an electronic case report form (eCRF). The eCRF should contain results of all the screening procedures, including patient history and demographics, imaging studies, bloodwork, filled-out questionnaires, operation note, and postoperative rounds during patient stay in the ICU and in the surgical ward.

### 2.9 Data collection, management, and analysis

#### 2.9.1 Data collection methods

All data will be collected prospectively using eCRFs designed for this trial. The reasons for withdrawal will be documented. The investigator will attempt to contact each participant at least 3 times during each follow-up window before declaring them lost for observation. The study exit form will be recorded in the eCRF. All prior data will be analyzed within the research.

#### 2.9.2 Data management

All patients will receive clarifications of all the study procedures, and will be able to discuss them with the primary investigator. All patient data will be handled according to the principles of doctor–patient confidentiality, the subjects will be anonymized and analyzed with individual identifier numbers transcribed into eCRF.

#### 2.9.3 Statistical methods

An intention to treat analysis will be performed. The quantitative variables will be described as means and standard deviations or as medians, interquartile range, and range, as appropriate. Categorical variables will be described in absolute numbers and percentages. The statistical analysis of the quantitative variables, with independent groups will be performed with the parametric Student *t* test, provided that its conditions for application are met. Otherwise, the nonparametric Mann–Whitney *U* test will be used. Statistical analysis for categorical variables will use the Pearson Chi-squared test or the Fisher exact test. Specifically, the above methods will be used to compare the 2 groups in terms of baseline characteristics in order to assess whether the randomization has been effective. Overall and recurrence-free survival rates will be estimated using the Kaplan–Meier method, and the log-rank test will be used to compare overall survival between the 2 groups. Differences between groups are considered significant at *P* = .05.

#### 2.9.4 Data monitoring

There is no data monitoring committee designated to this trial. Any adverse and serious adverse events will be immediately reported to the principal investigator and the primary sponsor. An interim analysis will be performed after 20 patients in each study group will have completed 3-month follow-up. All AEs and SAEs will be immediately reported to the local ethics committee, study director, and the sponsors.

### 2.10 Ethics and dissemination

#### 2.10.1 Research ethics approval

This study follows the Declaration of Helsinki on medical protocols and ethics; all documents of the trial have been approved by the local Ethics Committee of Sechenov University (reference No. 04-19, 03/06/2019).

#### 2.10.2 Protocol amendments

Any protocol amendments that may influence the conduct of the study, will be communicated to the local ethics committee and study director, and will be uploaded to clinical trials.

#### 2.10.3 Consent or assent

A member of the research team will obtain the consent form. All participants will be able to address their questions about the study to one of the members of the research team.

#### 2.10.4 Confidentiality

All patient data will be secured at the study site. No one apart from the members of the research team will have access to any patient data, including anonymized eCRFs with a coded ID, as well as filled out questionnaires.

#### 2.10.5 Declaration of interests

The authors declare they have no competing interests.

#### 2.10.6 Access to data

No one apart from the members of the research team will have access to the final trial dataset.

#### 2.10.7 Dissemination policy

Trial results will be e-mailed to all participants of the trial. Trial results will be disseminated to healthcare professionals via publication in a peer-reviewed scientific journal and by mass media, as well as conference papers to inform the public and stakeholders, and will be uploaded to the primary registry. We have no intention of granting public access to the full protocol, participant-level dataset, and statistical code.

## 3 Discussion

This is the 1st prospective randomized clinical trial comparing the efficacy and safety of 2- and 3-row-stapling techniques in LAR for rectal cancer. This study will primarily focus on short-term outcome assessment that will allow to gather preliminary information about the use of 3-row circular staplers in rectal cancer surgery.

The design of this study has certain limitations. Firstly, there is no definitive way to exclude all the preoperative and intraoperative risk factors of AL due to their large number and the presence of nonmodifiable risk factors, such as male gender, the need for preoperative chemo-/radio-/chemoradiotherapy, or tumor size. Moreover, broadening of exclusion criteria with regard to modifiable risk factors, such as tobacco use would hinder patient recruitment significantly, and thus would delay the acquisition of definite conclusions. Additionally, whether open or minimally invasive, TME ends with rectum transection below the inferior tumor margin using a linear cutter stapler followed by colorectal anastomosis formation with a circular stapler. Thus, the quality of both linear mechanical suture on the rectal stump and of the circular stapled anastomosis between the proximal colon and the distal rectal stump influence the integrity and healing rate of colorectal anastomosis. However, in the context of this study the distance of anastomosis from the anal verge is limited to LARs, which will help to achieve homogeneity of the study groups.

Another issue is the uncertainty of the definition of AL. In this protocol, we have referred to the definition of AL proposed by the International Study Group of Rectal Cancer describing it as a “communication between the intra- and extraluminal compartments owing to a defect of the integrity of the intestinal wall at that anastomosis between the colon and rectum or the colon and anus.”^[14]^ Because there are numerous definitions of AL, diagnostic approaches vary greatly between different studies. However, the algorithm of AL diagnosis outlined in this paper allows to detect both clinically significant and asymptomatic forms of AL ensuring adequate primary outcome assessment.

## Acknowledgment

The authors express their gratitude to Sechenov University administration for their support of this work.

## Author contributions

All listed authors fulfill the authorship criteria defined by the International Committee of Medical Journal Editors.^[17]^ IT, PT, and NN participated in the conception and design of the study, NN drafted the current manuscript, IT performed statistical analysis. IT, PT critically reviewed the manuscript, NN, IT were responsible for project administration and visual content. All authors read and approved the final manuscript.

**Conceptualization:** Inna A. Tulina.

**Data curation:** Inna A. Tulina.

**Formal analysis:** Inna A. Tulina.

**Investigation:** Nikita A. Nekliudov.

**Project administration:** Nikita A. Nekliudov.

**Resources:** Petr V. Tsarkov.

**Software:** Nikita A. Nekliudov.

**Supervision:** Petr V. Tsarkov, Inna A. Tulina.

**Validation:** Nikita A. Nekliudov.

**Visualization:** Nikita A. Nekliudov.

**Writing – original draft:** Nikita A. Nekliudov.

**Writing – review & editing:** Petr V. Tsarkov, Inna A. Tulina.

Petr V. Tsarkov orcid id: 0000-0002-7134-6821

Inna A. Tulina orcid id: 0000-0002-6404-389X

Nikita A. Nekliudov orcid id: 0000-0002-4291-5052
